# The development and characterisation of porphyrin isothiocyanate–monoclonal antibody conjugates for photoimmunotherapy

**DOI:** 10.1038/sj.bjc.6602517

**Published:** 2005-04-05

**Authors:** R Hudson, M Carcenac, K Smith, L Madden, O J Clarke, A Pèlegrin, J Greenman, R W Boyle

**Affiliations:** 1 Department of Chemistry, Clinical Biosciences Institute, University of Hull, Cottingham Road, Hull, East Yorkshire HU6 7RX, UK; 2 CRLC Val d’Aurelle-Paul Lamarque, Montpellier, France; 3 Postgraduate Medical School, Clinical Biosciences Institute, University of Hull, Cottingham Road, Hull, East Yorkshire HU6 7RX, UK

**Keywords:** photodynamic therapy, monoclonal antibodies, bioconjugation

## Abstract

A promising approach to increase the specificity of photosensitisers used in photodynamic therapy has been through conjugation to monoclonal antibodies (MAb) directed against tumour-associated antigens. Many of the conjugations performed to date have relied on the activated ester method, which can lead to impure conjugate preparations and antibody crosslinking. Here, we report the development of photosensitiser–MAb conjugates utilising two porphyrin isothiocyanates. The presence of a single reactive isothiocyanate allowed facile conjugation to MAb FSP 77 and 17.1A directed against internalising antigens, and MAb 35A7 that binds to a non-internalising antigen. The photosensitiser–MAb conjugates substituted with 1–3 mol of photosensitiser were characterised *in vitro*. No appreciable loss of immunoreactivity was observed and binding specificity was comparable to that of the unconjugated MAb. Substitution with photosensitiser had a minimal effect on antibody biodistribution *in vivo* for the majority of the conjugates, although a decreased serum half-life was observed using a cationic photosensitiser at the higher loading ratios. Tumour-to-normal tissue ratios as high as 33.5 were observed using MAb 35A7 conjugates. The internalising conjugate showed a higher level of phototoxicity as compared with the non-internalising reagent, using a cell line engineered to express both target antigens. These data demonstrate the applicability of the isothiocyanate group for the development of high-quality conjugates, and the use of internalising MAb to significantly increase the photodynamic efficiency of conjugates during photoimmunotherapy.

Photodynamic therapy (PDT) is a multicomponent anticancer treatment that involves the administration of a photosensitising drug and its subsequent activation by light of the appropriate wavelength (Macdonald and Dougherty, 2001). In the field of oncology, PDT is primarily used for the treatment of superficially localised tumours (Dougherty *et al*, 1998). In this context, the area illuminated is accessible, and treatment is associated with minimal toxicity, favourable tumour response and a good cosmetic outcome (Kubler *et al*, 1999). Photodynamic therapy has also been investigated for the treatment of deep-seated or disseminated malignancies (Biel, 1998); unfortunately, acute damage to surrounding normal tissue and treatment-related toxicity is often observed as a consequence of poor photosensitiser selectivity, and difficulties directing the light source to the tumour mass (Nseyo *et al*, 1998).

Several approaches have been investigated to increase the specificity of photosensitisers for cancerous tissues (Brasseur *et al*, 1999; Gijsens *et al*, 2000; Swamy *et al*, 2002). Of considerable interest has been the conjugation of photosensitisers to monoclonal antibodies (MAb) directed against tumour-associated antigens; this approach is often termed photoimmunotherapy (PIT). Conjugates of photosensitisers with MAb directed against a variety of targets have been produced including oncofoetal antigens (Carcenac *et al*, 1999), receptors for signal transduction pathways (Carcenac *et al*, 2001) and growth factor receptors (Hamblin *et al*, 2000).

A notable drawback in the development of well-characterised photosensitiser–MAb conjugates is the efficient conjugation of the photosensitiser to the antibody. The majority of current strategies for conjugation involve the use of photosensitisers bearing an activated ester functionality for conjugation to free amines within the MAb structure (Vrouenraets *et al*, 1999). Typically, photosensitisers in clinical trials are used. A limitation in such syntheses is the isolation of photosensitisers containing only one activated ester. Previously a derivative of *meta*-tetrahydroxyphenyl chlorin (*m*-THPC) has been synthesised bearing four activated esters on the *meta* positions of the meso phenyl rings. The monoesterified compound was then accessed through hydrolysis of this intermediate. However, the kinetics of the reaction are difficult to control and appreciable amounts of the fully hydrolysed and therefore unreactive photosensitiser are generated, as well as photosensitiser bearing two reactive esters (Vrouenraets *et al*, 1999). These impurities affect the stoichiometry of further reactions, and multiple reactive sites can cause antibody crosslinking during conjugation, leading to reduced yields and increasing aggregate formation, which can lead to nonspecific uptake. Alternatively, syntheses describing the activation of the carboxyl groups of monoaspartyl chlorin e6 (Npe6), often in conjunction with the use of polymeric carrier molecules, have been reported (Goff *et al*, 1991; Hamblin *et al*, 1996). The MAb and photosensitiser are attached to the carrier molecule, which usually acts to increase both photosensitiser loading and conjugate solubility. The photosensitiser is often used without purification and contains three carboxyl groups that may be functionalised, which could lead to crosslinking of the antibody or polymer. The use of a polymeric carrier may also affect the photophysics of the photosensitiser and can lead to nonspecific uptake.

In consideration of the above limitations, we have recently developed a series of porphyrin photosensitisers bearing a single reactive isothiocyanate group for conjugation to biomolecules (Clarke and Boyle, 1999; Sutton *et al*, 2002). Facile conjugation to bovine serum albumin was demonstrated with minimal non-specific binding (Sutton *et al*, 2002). We now report the use of photosensitiser isothiocyanates for conjugation to MAb. The previously developed photosensitisers were conjugated to MAb 35A7, FSP 77 and 17.1A. Monoclonal antibody 35A7 recognises and binds to carcinoembryonic antigen (CEA), which is overexpressed on colon adenocarcinomas and represents an antigen that is not internalised upon MAb binding. FSP 77 binds to the extracellular domain of the erb-B2 receptor, a receptor widely expressed on breast and ovarian cancers. The EpCAM epithelial antigen, to which MAb 17.1A binds, is upregulated in a variety of malignancies including colorectal carcinoma. FSP 77 and 17.1A are internalised following antigen binding. The conjugates were characterised *in vitro* and *in vivo*, and their potential for use in PIT *in vitro* was assessed using the SKOv3-CEA-1B9 cell line, which has been previously engineered to express both the CEA and erb-B2 antigens (Carcenac *et al*, 2001). Using this, we were able to compare directly the efficacy of the internalising conjugates with those that remain surface bound.

These data highlight the versatility of the isothiocyanate group, which allows conjugation to MAb without the generation of multiple reactive sites or *in situ* reactive intermediates. The enhanced cell killing efficiency of the internalising conjugates is also demonstrated, suggesting that such molecules should be the focus of future PIT studies.

## MATERIALS AND METHODS

### Photosensitisers

**Figure 1:**
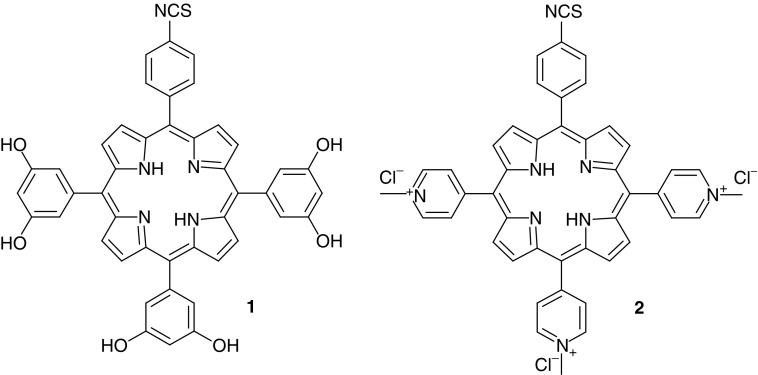
Chemical structure of porphyrin isothiocyanates.

The two water-soluble photosensitisers 5-(4-isothiocyanatophenyl)-10,15,20-tri-(3,5-dihydroxyphenyl)porphyrin (**1**) and 5-(4-isothiocyantophenyl)-10,15,20-tris-(4-*N*-methylpyridiniumyl)porphyrin trichloride (**2**) (Figure 1) were synthesised as described previously (Sutton *et al*, 2002). Briefly, condensation of 4-acetamidobenzaldehyde with either 3, 5-dimethoxybenzaldehyde or pyridine 4-carboxaldehyde under Adler–Longo conditions gave the corresponding monoacetamido porphyrins, following chromatographic separation. Subsequent acid hydrolysis yielded the analogous monoamino derivatives. Demethylation of the methoxy functionalities of porphyrin **1** using boron tribromide gave the hexa-hydroxylated analogue, which was subsequently treated with 1, 1′-thiocarbonyldi-2, 2′-pyridone (TDP) to yield the isothiocyanate. Porphyrin **2** was reacted with TDP to generate the isothiocyanate functionality prior to quaternisation with methyl iodide and anion exchange. The photosensitisers were rigorously purified following each synthetic step and stored at −20°C under a nitrogen atmosphere until further use.

### Monoclonal antibodies

The murine MAb 35A7 and FSP 77 (both IgG1) have been used previously for conjugation to tetrasulphonato aluminium phthalocyanine using the *in situ* generation of an active ester (Carcenac *et al*, 1999). MAb 35A7 binds to an epitope on CEA, which is overexpressed on foetal colon and colon adenocarcinomas (Khare *et al*, 2001). MAb FSP 77 binds to the extracellular domain of the erb-B2 receptor; this antigen is overexpressed on breast and ovarian cancers (Cuello *et al*, 2001). The MAb 17.1A (IgG2a; a generous gift from Professor S. Warnaar) is an anti-EpCAM antibody that has also been used previously as the targeting component of a photosensitiser immunoconjugate, which recognises a commonly overexpressed antigen on colorectal cancers that is internalised on binding (Hamblin *et al*, 2000).

### Radiolabelling and preparation of conjugates

The intact MAb 35A7 and FSP 77 were radioiodinated using the Iodogen method (1, 3, 4, 6-tetrachloro-3*α*, 6*α*-diphenylglycoluryl, Sigma, Poole, UK) to arrive at a final specific activity of 0.1 *μ*Ci of ^125^I or 1 *μ*Ci ^131^I per *μ*g of protein (1 *μ*Ci=37 kBq). Free radioiodine was separated from the MAb on a Sephadex G25 column (Amersham Biosciences, Uppsala, Sweden) eluting with 0.5 M sodium bicarbonate buffer (pH 9.2).

To a 1 ml solution of MAb, at a concentration of 1 mg ml^−1^ in 0.5 M bicarbonate buffer (pH 9.2), was added photosensitiser (PS) **1** in DMSO (50 *μ*l) or PS **2** in H_2_O (50 *μ*l). The photosensitisers were added separately at varying concentrations of either 20, 40 or 60 mol of photosensitiser per mole of MAb. The reaction vessels were agitated gently at room temperature for 1 h and protected from light. The conjugates were purified using Sephadex G25 columns (Amersham Biosciences, Uppsala, Sweden) equilibrated and eluted with PBS. The number of moles of photosensitiser conjugated per mole of antibody (degree of labelling, DOL) was calculated by spectroscopy. Briefly, the number of moles of photosensitiser per conjugate was calculated thus: *C*_photosensitiser_=*A*_420 nm_/*ε*. The MAb concentration (mg ml^−1^) was calculated using: MAb conc (mg ml^−1^)=*A*_280 nm_/1.3. This then allowed calculation of the number of moles of MAb per conjugate. The DOL was derived for each conjugate at all the initial molar ratios employed by division of the moles of photosensitiser by the moles of MAb.

### Cell lines

The LS174T human colon carcinoma cell line to which MAb 35A7 binds, SKOv3 human ovarian carcinoma cell line that MAb FSP 77 binds to and the hybrid cell line SKOv3-CEA-1B9 were available in our laboratories. The cell lines were grown under standard culture conditions. Cell lines were grown as monolayers at 37°C in an environment containing 5% CO_2_ in RPMI 1640 (Roswell Park Memorial Institute). This culture medium was supplemented with 10% (v v^−1^) foetal calf serum (FCS), 2 mM L-glutamine, 100 *μ*g ml^−1^ streptomycin, 100 *μ*g ml^−1^ penicillin and 0.25 *μ*g ml^−1^ amphotericin B (Gibco, Paisley, UK). Cells were subcultured regularly to ensure exponential growth. The human colorectal adenocarcinoma cell line Colo 320 (European Collection of Animal and Cell Cultures) was used in studies involving MAb 17.1A. The cells were grown in RPMI supplemented with 10% (v v^−1^) FCS, 2 nM L-glutamine, 100 *μ*g ml^−1^ streptomycin and 100 *μ*g ml^−1^ penicillin.

### Animals and tumour model

For experiments involving MAb 35A7, the human colon carcinoma cell line LS174T was implanted subcutaneously into the hind legs of Swiss nude mice (CRLC, Montpellier, France). SKOv3 cells were injected subcutaneously into the same animal model for studies using the MAb FSP 77. Mice were maintained and handled in accordance with the recommendations of the local animal care committee. The animals were allowed free access to food and water. All the *in vivo* experiments were performed in compliance with the French guidelines for experimental animal studies (Agreement No. B 34-172-27) and fulfil the UKCCCR guidelines for the welfare of animals in experimental neoplasia.

### Flow cytometry

Cells were removed from culture flasks with 5 mM EDTA in PBS. After washing cells were counted, resuspended in PBS/0.25% (w v^−1^) BSA and 2 × 10^5^ cells were added to each tube. The cells were labelled with 50 *μ*l (5 *μ*g ml^−1^) of primary antibody (conjugated/unconjugated/irrelevant) for 1 h at 4°C. After washing with PBS/0.25% (w v^−1^) BSA, cells were labelled with 50 *μ*l (10 *μ*g ml^−1^) of rabbit anti-mouse IgG–FITC (Serotec, Oxford, UK) for 1 h at 4°C. A further wash with PBS/0.25% (w v^−1^) BSA was performed before analysis of cells on a FACSCalibur flow cytometer (BD Biosciences, Oxford, UK).

### *In vitro* phototoxicity

This procedure was employed for analysis of the cytotoxicity of the conjugates of MAb 35A7 and FSP 77 using the SKOv3-CEA-1B9 cell line, and the conjugates of MAb 17.1A using the Colo 320 cell line. Cells in logarithmic growth phase were harvested and their concentration adjusted to 1 × 10^6^ cells ml^−1^. They were then incubated for 24 h in the dark with photosensitiser or conjugate at varying concentrations in the absence of serum. Following incubation, the cells were washed with DMEM (to eliminate unbound photosensitiser), resuspended and plated (8000 cells well^−1^) in quadruplicate into a 96-well plate. The plate was then irradiated with 10 J cm^−2^ of cooled and filtered red light (630 nm) delivered by a Patterson light system (Phototherapeutics Ltd, Albringham, UK: Patterson Lamp BL1000A, bandpass 630±15 nm filter). After irradiation, 5 *μ*l of FCS was added to each well and the plates (both irradiated and non-irradiated as a dark toxicity control) were incubated overnight.

The tetrazolium salt 3-4, 5-dimethylthiazol-2-yl)-2, 5-diphenyltetrazolium bromide (MTT) assay was performed 18–24 h postirradiation. Briefly, 10 *μ*l of MTT solution (5 mg ml^−1^ PBS, MTT Thiazoyl blue, Sigma Cat. No. M-5655) was added to each well. The plates were then returned to the incubator for 4 h or until sufficient colour developed. When this was accomplished, 150 *μ*l of isopropanol-HCl (0.04 *N*) was added to each well to stop the reaction. The cells were lysed mechanically, and the blue formazan crystals produced by the mitochondria of living cells were dissolved by vigorous pipetting. The plates were then read at 570 nm using a Bio-Tek Elx 800 plate reader. The percentage of cell survival was calculated in proportion to the number of cells incubated without photosensitiser.

### *In vitro* binding

To measure the *in vitro* binding and nonspecific binding of the radiolabelled conjugates of MAb 35A7 and FSP 77, purified antigen (CEA or erb-B2) was used immobilised on sepharose. The procedure was performed in quadruplicate. An irrelevant sepharose-bound antigen (Px) was used to assay any non-specific antigen binding of the conjugates. Consequently, 1 *μ*g of purified sepharose-bound antigen was added per tube. A control tube containing sepharose without antigen was used in each experiment to assay any non-specific binding to the sepharose alone. To each of these tubes was added 20 ng of either conjugate or unconjugated antibody as a 200 *μ*l solution of BSA–PBS buffer containing 1% (v v^−1^) normal mouse serum. The total radioactivity of each tube was measured for 1 min. The tubes were then incubated overnight at room temperature with gentle agitation. After incubation, the tubes were washed with 2 ml BSA–PBS (1 mg ml^−1^) and centrifuged for 5 min at 3000 r.p.m. The supernatant was removed by aspiration and the washing procedure repeated. Once complete, the radioactivity of the tubes was again measured for 1 min. The percentage *in vitro* binding or nonspecific binding was calculated using the following equation: percentage binding=(*γ* after incubation/*γ* before incubation) × 100.

### *In vivo* biodistribution

Biodistribution studies were performed in Swiss nude mice bearing subcutaneously implanted xenograft (LS174T and SKOv3). At 24 h before injection, 0.5 ml of pure Lugol's iodine solution was added to the drinking water to block the thyroid glands of the mice. A mixture of ^125^I (conjugates) and ^131^I (unconjugated Mab) (each at a specific radioactivity of 1 *μ*Ci/10 *μ*g MAb) was intravenously injected into three mice per group when the tumours had reached a diameter of 3–5 mm. At 24 h after injection, the mice were killed by CO_2_ inhalation. Each mouse was dissected and the organs, body parts and blood placed in separate preweighed tubes. All the tubes were weighed separately before and after the addition of the body parts and the difference between empty and filled tubes calculated. The ^125^I and ^131^I radioactivities of each tube were measured in a dual-channel scintillation counter (Cobra II auto-gamma, Packard). ^131^I activity (14%) was subtracted from ^125^I radioactivity to compensate for overlap of ^131^I-*γ*-rays in the ^125^I window. Radioactivity levels are expressed as the percent of injected dose per gram of tissue (% ID g^−1^). The tumour-to-normal tissue ratios were also calculated.

## RESULTS

### Conjugation and flow cytometric analysis of conjugate binding

**Figure 2:**
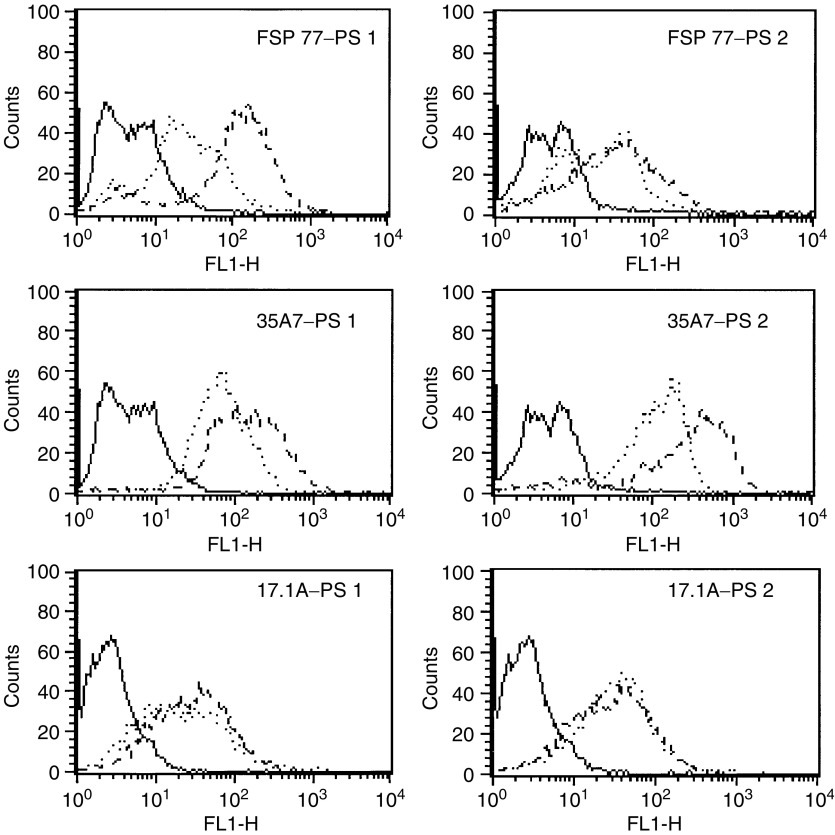
Indirect flow cytometric analysis of conjugate binding (bold, irrelevant control; dashed, unconjugated MAb; dotted, porphyrin conjugates).

The two photosensitisers were conjugated to MAb 35A7, FSP 77 and 17.1A as described, using an initial molar ratio of 20. The purified conjugates had a DOL between 1.5 and 2.0. Following conjugation, the reactivity of the MAb–porphyrin conjugates was compared to that of the unconjugated MAb using the appropriate antigen-positive cell lines by flow cytometry (Figure 2).

The binding of the conjugates prepared using MAb 35A7 and FSP 77 were analysed using the SKOv3-CEA-1B9 cell line. This cell line had originally been engineered from the erb-B2-expressing cell line SKOv3 to simultaneously express CEA as well as erb-B2 (Carcenac *et al*, 2001). The conjugates prepared using MAb 17.1A were evaluated using the Colo 320 cell line. The conjugate binding (dotted lines) to the respective cell lines is comparable to that of the unconjugated MAb (dashed lines) for each MAb and cell line employed. Conjugating PS **1** and PS **2** to MAb 35A7 and FSP 77 resulted in slightly diminished binding to the SKOv3-CEA-1B9 cell line (top four histograms), although the conjugates displayed positive binding with respect to the irrelevant control. The FSP 77–PS **1** conjugate displayed a more pronounced loss of binding, although it was still positive with respect to the control. No apparent loss of binding was observed using the conjugates of MAb 17.1A on Colo 320 cells.

### *In vitro* cytotoxicity

The photocytotoxicity of the photosensitisers and conjugates was assayed by the MTT assay using the SKOv3-CEA-1B9 cell line (35A7 and FSP 77) and the Colo 320 cell line (17.1A). Use of SKOv3-CEA-1B9 cells allowed direct comparison between internalising and non-internalising conjugates on the same cell line. The IC_50_ values for the photosensitisers and conjugates were measured to allow for comparative assessment of the effects of MAb conjugation on phototoxicity and are presented in Tables 1 and 2. The photosensitisers and conjugates were not toxic to the cell lines in the absence of light (data not shown).

The PS **1**–17.1A conjugate caused almost five times more cell growth inhibition than the free photosensitiser. However, coupling PS **2** to the MAb inhibited cell growth by 90% at a concentration of only 2.2 *μ*M, in comparison to the free photosensitiser, which required 29.0 *μ*M to cause just 50% cell growth inhibition. The non-internalising PS **1**–35A7 conjugate was less effective against the SKOv3-CEA-1B9 cell line than the free photosensitiser. In contrast, the internalising PS **1**–FSP 77 conjugate gave an IC_50_ value 15-fold less than that of the free photosensitiser. A similar trend is observed with the conjugates of PS **2**, although both the conjugates in this case were more phototoxic than the unconjugated photosensitiser. Again, the internalising PS **2**–FSP 77 conjugate led to greater cell growth inhibition, having an IC_50_ value five-fold lower than the non-internalising conjugate and 14 times less than the free photosensitiser. Overall, conjugating the photosensitisers to MAb increases their potential for PIT *in vitro*, although internalising conjugates are clearly the most efficient.

### Increased loading ratios and *in vitro* antigen binding

Having established a protocol for conjugating photosensitiser isothiocyanates to MAb, demonstrated retention of MAb binding by FACS and enhanced photodynamic efficiency *in vitro*, the effect of increasing the porphyrin loading of the MAb was examined to see if more potent derivatives could be constructed. The conjugates were then characterised further in an *in vivo* biodistribution study. Since limited quantities of MAb 17.1A were available and MAb FSP 77 represents an MAb that binds to an internalising receptor, the study was completed using only MAb 35A7 and FSP 77. To allow for comparative biodistribution studies using the MAb conjugates and unconjugated antibody, the antibodies were radiolabelled with either ^125^I (conjugates) or ^131^I (unconjugated antibodies). Repeat conjugations were performed using initial molar ratios of 20, 40 and 60 and the conjugates were purified as before. The overall DOL of the antibodies with the photosensitisers was measured spectroscopically and is presented in Table 3.

Increasing the initial molar ratio generally led to minor increases in the DOL of the conjugates with photosensitiser. However, the DOL of the PS **1**–FSP 77 conjugate decreased as the initial molar ratio increased. This was thought to be a consequence of the photosensitiser diminishing the solubility of the MAb in PBS at higher loading ratios. This also corresponded to a reduced recovery of the conjugate from the Sephadex G25 column, an effect that has been reported elsewhere (Vrouenraets *et al*, 2000). The conjugates synthesised using PS **2** and MAb 35A7 were recovered quantitatively. Clearly, when conjugating PS **1** with MAb FSP 77 at initial molar ratios of 40 and 60, only the least substituted MAb is soluble enough to pass through the column.

The immunoreactivity of the conjugates *in vitro* was compared to that of the unconjugated antibodies in a direct binding assay using CEA and erb-B2 immobilised on sepharose. An irrelevant antigen (Px) was used to assay any nonspecific binding. The conjugate binding to the respective antigens was not statistically different to that of the unconjugated antibodies (data not shown) and the nonspecific binding was less than 5% in each case.

### *In vivo* biodistribution

**Figure 3:**
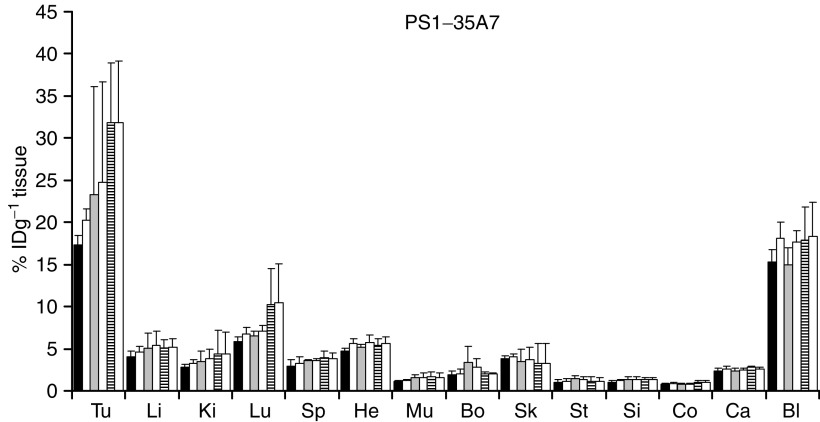
Biodistribution of unconjugated MAb control (open bars) and PS **1**–35A7 conjugate at the following DOL: 1.4 (black bars), 1.6 (grey bars), 2.0 (crossed bars). Tu, tumour; Li, liver; Ki, kidney; Lu, lungs; Sp, spleen; He, heart; Mu, muscle; Bo, bone; Sk, skin; St, stomach; Si, small intestine; Co, colon; Ca, carcass; Bl, blood. The values were obtained 24 h postinjection. Values are expressed as the mean±s.d. calculated from the three mice bearing the LS174T xenograft in each conjugate group. The conjugates were compared to unconjugated MAb in three separate experiments.

For biodistribution analysis of the ^131^I-MAb 35A7 and ^125^I-MAb 35A7–porphyrin conjugates, the LS174T cell line was subcutaneously implanted into the hind legs of Swiss nude mice. To assay the biodistribution of the ^125^I-MAb FSP 77 conjugates and ^131^I-labelled free antibody, SKOv3 cells were injected into the hindquarters of the same animal model. The biodistribution of the conjugates of each photosensitiser at differing degrees of labelling was compared to that of the unconjugated antibody for each conjugate subset. Figure 3 shows the biodistribution profile for the PS **1**–35A7 conjugate at different DOL. The biodistribution of each conjugate was compared to the unconjugated antibody in each experiment. The % ID g^−1^ tumour and tumour-to-normal tissue ratios for representative peritumoral tissues for all the conjugates at each DOL are shown in Tables 4 and 5. The conjugates prepared using PS **1** have tumour uptake values comparable to those of the unconjugated MAb 35A7 (Figure 3) and FSP 77. Increasing the DOL of the conjugates using PS **2** is associated with a decreased uptake in tumour, in comparison to the unconjugated MAb. For example, using PS **2**–35A7 conjugates with DOL of 1.6, 2.3 and 2.5 gave tumour uptake values of 23.9, 17.4 and 12.9% ID g^−1^ tissue, respectively.

Concurrent with biodistribution studies using a similar photosensitiser (Vrouenraets *et al*, 2000), it is evident that increased loading with PS **2** decreases the residence time of the MAb in the blood, resulting in reduced tumour uptake. The tumour-to-normal tissue ratios for the MAb 35A7 conjugates are significantly higher than those observed using the MAb FSP 77 conjugates. The tumour/blood ratios for the MAb 35A7 conjugate are moderate but similar to values reported elsewhere (Carcenac *et al*, 1999). The values for the MAb FSP 77 conjugates are slightly lower. This may reflect an increased MAb to tumour transport time using this antibody. Overall, the ratios for both antibody conjugates were still significantly higher than typical ratios reported using common photosensitisers (Boyle and Dolphin, 1996). Tumour-to-colon ratios for the PS **1**–35A7 (DOL, 2.0) and PS **2**–35A7 (DOL, 1.6) conjugates were 33.3 and 33.5, respectively. Since the conjugates are directed against CEA, which is overexpressed on colorectal cancers in comparison to that on normal tissues, these values suggest the potential for more selective photosensitiser delivery to this tumour type.

## DISCUSSION

The use of MAb to increase the specificity of photosensitisers for malignant tissues was first reported by Mew *et al* (1983). Alternative methods of conjugating to MAb using better defined photosensitisers and polymeric carriers were subsequently developed (Jiang *et al*, 1990; Goff *et al*, 1991). However, these conjugate preparations often contained significant amounts of impurities. A more refined method for conjugation to polymeric cationic and anionic carriers was later developed by Hamblin *et al* (1996) and was used successfully *in vitro* (Del Governatore *et al*, 2000) and *in vivo* (Duska *et al*, 1997). Within these studies, a chlorin e6-NHS ester is used without purification prior to attachment to the macromolecule. Also polycationic macromolecules can increase the rate of nonspecific cell-mediated endocytosis (Shen and Ryser, 1978). Other methodologies, also relying on activated ester groups, have involved direct attachment of the photosensitiser to the MAb (Vrouenraets *et al*, 1999, 2001). The photosensitisers *m*-THPC and [AlPc(SO3H)]_4_ with a trifluorophenyl (TFP)-ester group were obtained by hydrolysis of the corresponding *tetra*-TFP-ester intermediates. However, the procedure led to preparations containing appreciable amounts of fully hydrolysed by product. Savellano and Hasan (2003) later developed a purified monofunctionalised BPD-MA derivative and used a 50% DMSO/50% water system for conjugation. This procedure yielded well-characterised conjugates, although the solvent system is unlikely to be universally tolerated by MAb.

In the present study, we have investigated the use of photosensitiser isothiocyanates for conjugation to MAb. The photosensitisers were synthesised according to a previously published method (Clarke and Boyle, 1999). This synthesis yields pure photosensitiser, which unambiguously presents a single amino reactive group. Efficient conjugation to BSA, with negligible non-specific binding, has been previously demonstrated (Sutton *et al*, 2002). In a continuation of the study, the photosensitisers were conjugated with MAb 35A7, FSP 77 and 17.1A. Conjugations were performed under ambient conditions and the procedure allowed direct attachment of the photosensitisers to the MAb, with no prior modification of the antibodies. In this context, the isothiocyanate group represents a superior functionality for conjugation of photosensitisers to MAb. Retention of antigen binding was demonstrated using flow cytometry. The *in vitro* photoinduced cell inhibition of the conjugates was compared to the unconjugated photosensitisers. Conjugation to MAb 17.1A led to a significant decrease in the concentration of photosensitiser required for growth inhibition of the Colo 320 cell line as compared with free photosensitiser. Comparative analysis of the non-internalising 35A7 conjugates and the internalising FSP 77 conjugates was performed using the SKOv3-CEA-1B9 cell line. The non-internalising conjugates using MAb 35A7 were generally more effective than the free photosensitiser and the internalising MAb FSP 77 conjugates were significantly more active than the 35A7 conjugates or the free photosensitiser. The PS **1**–FSP 77 conjugate gave an IC_50_ value 16 times less than that of the unconjugated photosensitiser. Concurrent with results from previous studies (Carcenac *et al*, 2001), the use of internalising MAb in PIT offers advantages over conjugates of non-internalising MAb or free photosensitisers.

Radiolabelled conjugates were then prepared for use in *in vivo* biodistribution studies. MAb 35A7 and FSP 77 were chosen to represent MAb that are non-internalising and internalising, respectively. The biodistribution of the ^125^I-labelled conjugates was then compared to that of the ^131^I-labelled MAb in Swiss nude mice subcutaneously implanted with the appropriate antigen-expressing cell lines. The MAb conjugates with PS **1** had biodistribution values comparable to the unconjugated antibodies. A high uptake in the blood was observed with MAb FSP 77. The least substituted conjugates of PS **2** and the two MAb had tumour uptake values similar to the unconjugated antibodies. When the substitution of the MAb with the photosensitiser increased, the uptake in the tumour and other organs decreased significantly. This is consistent with a report by Pardridge *et al* (1998) who found that increasing an MAb cationic charge reduced its serum half-life to 5% of that of the unmodified antibody. The data from the biodistribution studies clearly demonstrates retention of MAb pharmacokinetics following substitution with PS **1** and to a lesser degree with PS **2**. The observed tumour/normal tissue ratios using representative surrounding tissue for colon carcinomas were exceptionally high at 33.5 for the MAb 35A7 PS **2** conjugate. Tumour/normal tissue ratios between 2 and 4 have previously been reported for unconjugated photosensitisers (Boyle and Dolphin, 1996). Indeed, if tumour/normal tissue ratios could be significantly improved, PDT may play an important role in destroying early stage metastasis after surgical resection of large tumour masses. In conclusion, we have developed a convenient and widely applicable strategy for the conjugation of photosensitisers to antibodies that offers several advantages over methods currently employed for conjugation. The conjugates retained antigen binding and had pharmacokinetics comparable to the unconjugated antibodies. The use of conjugates of internalising antibodies offered clear advantages over free photosensitisers in PIT studies *in vitro*, in terms of both specificity and cytotoxicity, and hold promise for clinical use. Experiments assessing the phototoxicity of the conjugates *in vivo* are currently underway in our laboratories.

## Figures and Tables

**Table 1:** IC_50_ values (*μ*M) for the free PS **1** and PS **2** and MAb–17.1A conjugates to the Colo 320 cell line

	**IC_50_ value**
PS **1**	11.5±2.1
PS **1**–17.1A	2.50^a^
PS **2**	29.0±4.2
PS **2**–17.1A	2.2^a^^,^^b^

MAb=monoclonal antibodies; PS=photosensitisers. Values expressed as the mean±s.d. of three experiments.

a S.d. could not be determined in these cases due to the limited availability of MAb 17.1A.

b LD_90_ value.

**Table 2:** IC_50_ values (*μ*M) for the free PS **1** and PS **2** and MAb conjugates to the SKOv3-CEA-1B9 cell line

	**IC_50_ value**
PS **1**	68.0±18.0
PS **1**–35A7	No IC_50_
PS **1**–FSP 77	4.2±0.9
PS **2**	24.0±2.1
PS **2**–35A7	10.0±3.0
PS **2**–FSP 77	1.7±1.1

MAb=monoclonal antibodies; PS=photosensitisers. Values expressed as the mean±s.d. of three experiments.

**Table 3:** MAb–PS conjugate DOL at differing initial molar ratios

	**MAb 35A7**		**MAb FSP 77**	
	**DOL**			
**Initial molar ratio**	**PS 1**	**PS 2**	**PS 1**	**PS 2**
20	1.4	1.6	2.8	1.6
40	1.6	2.3	2.3	2.0
60	2.0	2.5	1.6	2.0

MAb=monoclonal antibodies; DOL=degree of labeling; % ID g^−1^=percent of injected dose per gram of tissue; PS=photosensitisers.

**Table 4:** % ID g^−1^ of tumour and tumour/normal tissue ratios for MAb 35A7 conjugates

		**Ratio**			
**Conjugate+DOL**	**% ID g^−1^ tumour**	**Tumour/blood**	**Tumour/muscle**	**Tumour/colon**	**Tumour/small int.**
PS **1** (1.4)	17.3±1.1	1.1±0.1	15.6±0.7	22.1±1.7	16.7±1.3
PS **1** (1.6)	23.3±12.8	1.5±0.7	16.8±10.0	29.4±15.9	18.8±12.3
PS **1** (2.0)	31.8±±7.0	1.8±0.1	18.5±2.8	33.3±5.0	23.0±3.2
PS **2** (1.6)	23.9±3.4	2.1±0.2	24.9±3.4	33.5±3.8	26.3±5.2
PS **2** (2.3)	17.4±6.2	1.9±0.7	19.8±6.0	25.1±10.3	14.8±12.2
PS **2** (2.5)	12.9±4.2	1.9±0.6	16.6±5.6	18.7±5.3	13.4±4.7

% ID g^−1^=percent of injected dose per gram of tissue; MAb=monoclonal antibodies; DOL=degree of labeling; PS=photosensitisers. Values were obtained 24 h postinjection (values are the means of tumour/normal ratios and % ID g^−1^ tumour±s.d. calculated from the three mice bearing the LS174T xenograft in each conjugate group).

**Table 5:** % ID g^−1^ of tumour and tumour/normal tissue ratios for MAb FSP 77 conjugates

		**Ratio**			
**Conjugate+DOL**	**% ID g^−1^ tumour**	**Tumour/blood**	**Tumour/muscle**	**Tumour/colon**	**Tumour/small int.**
PS **1** (2.8)	6.4±4.7	0.6±0.1	6.1±0.4	9.1±0.4	7.2±0.6
PS **1** (2.3)	7.4±2.2	0.6±0.2	5.6±0.2	10.6±2.7	5.3±2.2
PS **1** (1.6)	6.8±0.2	0.9±0.7	3.35±2.8	7.5±0.3	3.75±2.2
PS **2** (1.6)	8.5±2.1	1.06±0.1	9.2±6.3	13.8±8.7	10.7±6.4
PS **2** (2.0)	5.3±0.2	0.6±0.2	5.2±0.5	8.6±4.1	5.6±0.9
PS **2** (2.0)	4.2±0.1	0.6±0.1	4.4±0.4	7.0±0.5	5.5±0.1

% ID g^−1^=percent of injected dose per gram of tissue; MAb=monoclonal antibodies; DOL=degree of labeling; PS=photosensitisers. Values were obtained 24 h postinjection (values are the means of tumour/normal ratios and % ID g^−1^ tumour±s.d. calculated from the three mice bearing the SKOv3 xenograft in each conjugate group).
